# When expectation meets experience: A qualitative analysis of serial interviews with adults before and after autism assessment

**DOI:** 10.1177/13623613251384436

**Published:** 2025-10-30

**Authors:** Maria Downey, Juwayriyah Nayyar, Suzanne Guerin, Cliodhna O’Connor

**Affiliations:** 1 University College Dublin, Ireland

**Keywords:** adulthood, adults, assessment, autism, diagnosis, interviews, lived experience

## Abstract

With growing numbers of adults seeking and receiving autism diagnoses, understanding subjective experiences of this process is crucial for sensitive policy and practice. The route to diagnosis can be long and circuitous, yet most evidence on adults’ experiences of diagnosis relies on retrospective reports collected at a single point in time. The current study explores lived experiences of the diagnostic journey through serial qualitative interviews conducted in the weeks before and after adults’ autism diagnosis. Fourteen adults participated in online interviews over videoconferencing or email. Thematic analysis of the data suggested the diagnostic process could be characterised according to three themes. *Journey to Self-Discovery* identified the pursuit of diagnosis as rooted in a drive for self-understanding, propelled by anticipated benefits that were partially realised, and emotionally complex at all stages. *Challenges with Navigating the Diagnostic Process* highlighted how the process of acquiring and adjusting to a diagnosis was shaped by issues of resource access, system deficiencies, social inequalities and cognitive overwhelm. *Isolation to Advocacy* revealed how journeys that began in independent self-initiative evolved over time into community participation and advocacy. Providing original evidence of how lived experiences of adult autism diagnosis evolve across time, the current study offers valuable context for adults pursuing assessment, professionals performing assessments and policy-makers designing adult autism services.

**Lay abstract:**

Increasing numbers of people are receiving autism diagnoses in adulthood. Understanding their firsthand experience of diagnosis is crucial for improving supports for adults undergoing autism assessment. This study conducted interviews with adults at two time-points: one interview during the six weeks before their assessment, and a second interview within six weeks after their assessment. Fourteen people volunteered to participate in the study through videocall or email. The interview transcripts were analysed using a process called thematic analysis, with specialist software used to find patterns across people’s experiences. The analysis suggested that adults appreciated the role of diagnosis in helping develop a better understanding of themselves. Nevertheless, people had mixed emotions both leading up to the assessment and after their autism diagnosis. Adults experienced many challenges in arranging and undergoing the autism assessment. However, the process of getting a diagnosis helped many to move from feeling isolated to feeling part of a wider community. These findings will be useful to adults at different stages of the diagnosis process, and for professionals and policy-makers developing adult autism services.

Since its emergence as a diagnostic category in the mid-20th century, autism has largely been considered a childhood issue. The perception of autism as a transitory disorder, which children ultimately outgrow, has left autistic adults under-served and under-researched. However, growing appreciation of the lifelong nature of autism has seen increasing numbers seeking and acquiring diagnoses in adulthood (G. Russell et al., 2022). This study explores lived experiences of this diagnostic journey, through serial qualitative interviews with adults performed before and after an autism assessment.

The number of adults who hold a diagnosis of autism has increased dramatically since the turn of the century (Grosvenor et al., 2024; G. Russell et al., 2022). Many such individuals have acquired their diagnosis in adulthood (Huang et al., 2020). As late diagnoses can bring numerous social and emotional challenges (O’Connor et al., 2022), this cohort has distinct psychological and pragmatic needs. However, the quality of adult autism services is highly variable across jurisdictions (Scattoni et al., 2021), with common challenges including long wait-times for assessment and limited post-diagnosis supports (Nayyar et al., 2025; Norris et al., 2025; Wigham et al., 2023).

Understanding firsthand experiences of adult autism assessment is crucial for improving supports throughout the diagnostic process. Recent systematic reviews identify a growing body of research exploring lived experiences of adult autism diagnosis (Huang et al., 2020; Kiehl et al., 2024; Nayyar et al., 2025). Taken as a whole, this literature finds that autistic adults characterise their pre-diagnosis lives in terms of feeling ‘different’, isolated and misunderstood. Once suspicion of autism’s relevance arises, barriers to assessment include availability of appropriate specialists, financial challenges and difficulties navigating healthcare systems. Experiences of the assessment process itself varies, with some reporting difficulties communicating with professionals or subjection to juvenile- or masculine-oriented diagnostic practices. Post-diagnostic experiences are characterised by variable access to supports, and complex emotional reactions that mingle grief and regret with relief and hope. Ultimately, benefits to self-understanding and self-acceptance, and development of new communities and identities, seem to convince most informants that acquiring the diagnosis was worthwhile (Huang et al., 2020; Kiehl et al., 2024; Nayyar et al., 2025).

A major limitation of the literature on lived experiences of autism diagnosis is that the vast majority of data are retrospective, that is, provided after the assessment process has completed (Huang et al., 2020; Kiehl et al., 2024; Nayyar et al., 2025). These accounts provide important insight into the multidimensional consequences of diagnosis and post-diagnosis reframing of biographical narratives (Nayyar et al., 2025). However, retrospective reports, which may be collected several years from the diagnosis episode, are also subject to deficiencies of memory and cognitive biases (Bernard et al., 1984). For instance, participants may not recall certain concerns or expectations they had leading up to the diagnosis, if these were not realised in subsequent experiences. Retrospective data may therefore give an incomplete account of the holistic process of diagnosis, a journey which can take many months and involve numerous detours and diversions.

Attaining direct insight into pre-diagnosis experiences is important because this context involves a growing number of people, who – due to service deficiencies in many jurisdictions (Scattoni et al., 2021) – may remain in-waiting for considerable lengths of time. In addition, by definition it involves a wider cohort of adults than the post-diagnosis period, since some individuals will not progress to autism diagnosis (A. J. Russell et al., 2016). With considerable research enlightening post-diagnosis support requirements (Norris et al., 2025), the needs of people awaiting assessment remain unclear. Furthermore, pre-diagnosis experiences may shape subsequent responses to diagnosis in important ways. For instance, expectations of assessment as a therapeutic exercise, or that diagnosis will lead to targeted supports, could provoke disillusionment or despair if disconfirmed by later experience.

The current study advances understanding of lived experiences of adult autism diagnosis through analysis of serial interviews conducted with adults pre- and post-assessment. The analysis considers how the expectations and emotions experienced while awaiting assessment relate to subsequent responses to assessment outcomes. By incorporating multiple time-points, the study aims to provide fresh insight into adult autism diagnosis as a journey, rather than singular event.

## Method

### Design

Semi-structured online interviews were conducted with participants at two time-points: one within the six-week period before a scheduled autism assessment, and one in the six weeks after assessment. Serial interviews were chosen for several advantages they offer, including the ability to observe evolving perspectives, explore the implications of a focal event, and develop interpersonal rapport that facilitates rich, nuanced data (Murray et al., 2009; Read, 2018). The study received ethical approval from University College Dublin.

### Participants

Adults awaiting autism assessment were recruited via adverts on autism-specific websites and social media pages. Inclusion criteria stipulated a scheduled autism assessment, no previous childhood/adulthood autism diagnosis and ability to participate in two English-language online interviews. Fourteen adults opted into the study: 11 women and 3 men. Most (*n* = 9) were aged between 20 and 39 years, with five between 40 and 59 years. Participants were based in North America (*n* = 8), Europe (*n* = 5) or Australia (*n* = 1). Ethnicity or socio-economic data were not collected. Only one participant (from the United States) reported accessing an assessment via free/public healthcare systems: 13 paid for private assessments, with 4 reporting some contribution from health insurance. Prompts for seeking assessment included witnessing friends/family members get diagnosed (*n* = 6), clinical advice during treatment for other mental health issues (*n* = 5) and online research/tests (*n* = 3). Waiting times for assessment ranged from 3 weeks to 2.5 years.

### Procedure

After emailing to express interest, potential participants were sent an information sheet and consent form for electronic signature. To accommodate diverse communication preferences, participants could choose to complete semi-structured interviews over videocall (Zoom) or email. Most opted for Zoom interviews, with three participants completing both interviews over email and one participant completing one interview over email (pre-assessment) and one over Zoom (post-assessment). Participants’ assessment appointment dates were recorded in the first wave of interviews, with the researcher following up once those dates had elapsed to schedule post-assessment interviews. Two participants did not complete post-assessment interviews. Pre-assessment interview schedules queried: the events/motivations that led to seeking assessment, feelings about getting assessed, hopes about assessment outcomes, anticipated emotions on getting a diagnosis, and experiences sharing assessment information with others. Post-assessment interviews addressed: participants’ feelings about the diagnostic outcome, experiences of the assessment process, personal meanings around the diagnosis, changes to one’s life, other people’s reactions, and post-diagnosis supports. Interviews were recorded and transcribed with identifying data omitted. Pre-diagnosis interview transcripts ran for an average of 2590 words and post-diagnosis transcripts 3675 words. Once transcripts were completed, recordings and contact details were deleted and participants were assigned numerical identifiers.

### Analysis

**Figure 1.**
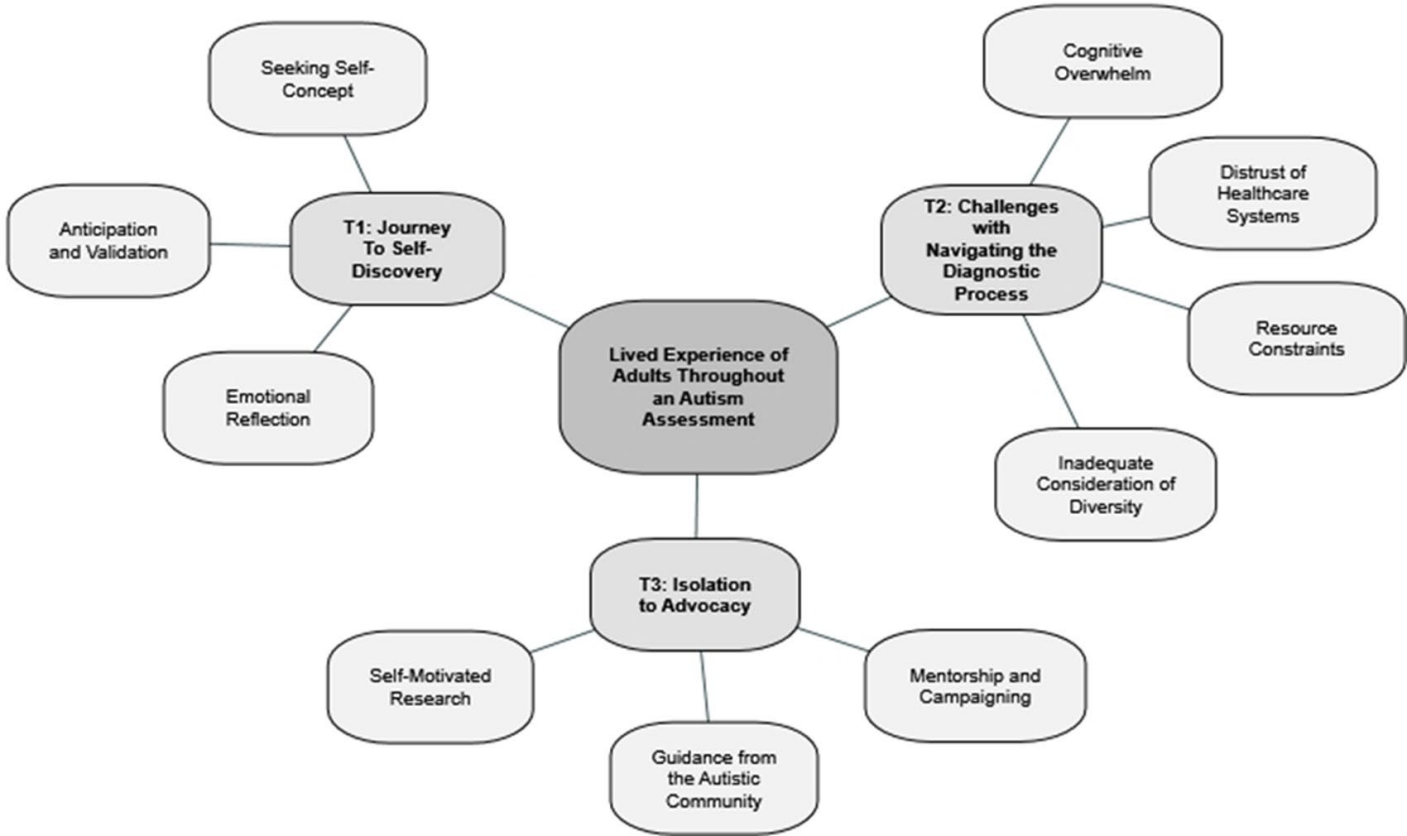
Thematic network.

Interview transcripts were analysed using the specialist software NVivo 20. To ensure sensitivity to the evolution of perspectives over time, individuals’ pre- and post-assessment interviews were analysed together as a single case. Interviews were analysed via inductive thematic analysis (Joffe, 2012). First, transcripts were read through several times, with notes made to record evolving patterns. These notes were iteratively developed into a coding frame containing 25 codes that captured the semantic meaning of the data. Through consideration of the content classified under each code, similar codes were grouped into 10 subthemes, which were in turn organised into 3 overarching themes (Figure 1).

While certain approaches to thematic analysis avoid assessment of coding reliability (Braun et al., 2018), other traditions encourage it as a means to promote consistency of coding and provoke reflexive dialogue across a research team (Joffe, 2012). Aligning with this approach, an additional person external to the project independently coded data from four transcripts using the final coding frame. Guided by O’Connor and Joffe’s (2020) procedure for assessing intercoder reliability, coding patterns were compared by calculating Cohen’s κ values through NVivo’s Coding Comparison Query function. Most (68%) codes met accepted criteria for agreement (κ > 0.4), four codes showing ‘fair’ agreement (κ = 0.21–0.4) were tightened in definition, and three codes with κ < 0.2 were dropped before final coding (O’Connor and Joffe, 2020). In line with a critical realist approach, the intent of intercoder reliability assessment was not to isolate a single ‘correct’ analysis of the data, but to allow the team to confirm their interpretations of codes were consistent and provide a platform for elaborating code definitions.

### Positionality

The research team included individuals with lived, familial, scientific and professional experience with autism, and the research was designed in consultation with an autistic adult and clinician who specialises in adult autism assessment. This multi-perspectival input helped shape inclusive recruitment materials, flexible interview protocols and context-sensitive interpretations of the data. In the analysis, efforts were made to privilege participants’ own language and framing of their experiences. Researchers kept reflexive notes throughout data collection and analysis to document their evolving understandings and any assumptions brought to the interpretation.

## Results

All 12 participants who returned for post-assessment interviews received an autism diagnosis. Figure 1 depicts the network of themes and subthemes produced by the analysis. The three themes are outlined below with illustrative quotes. Quotes are ascribed to numerical participant identifiers, with ‘a’ and ‘b’ indicating pre- and post-assessment interviews, respectively.

### Theme 1: Journey to Self-Discovery

This theme described the self-development experienced by adults during the diagnostic process.

#### Seeking self-concept

All participants had proactively initiated their diagnostic journey due to a desire to clarify and develop their self-concept. Conscious of their idiosyncrasies and deviation from social norms, participants in the pre-assessment phase expressed a strong desire for self-explanation: ‘answers, anything [. . .] I just need answers’ (P07a) to help ‘understand myself better’ (P12a). A major motivation for their request for assessment was a wish to understand their own thoughts, emotions and behaviour: ‘what’s been kind of driving some of the behaviour and hear what I have experienced internally’ (P14a). Participants anticipated that the explanatory value of an autism diagnosis would outweigh any connotations of deficit or disorder:

I just needed something wrong with me. That’s what I felt. (P02a)

I will soon have answers for things that have plagued me my whole life. (P11a)

All pre-assessment participants expressed strong suspicion they were autistic, with some ‘already convinced’ (P14a) of its veracity. However, others described self-doubt in their intuition: ‘a tendency to, like, gaslight myself and say that, like, maybe this thing that I’m doing isn’t really autism’ (P06a). Thus, uncertainty about the potential diagnosis’ veracity shaped the pre-assessment period:

there’s that kind of imposter syndrome in me where I am pretty sure, but there’s still that, like niggling in the back of my mind like, am I really? Am I just faking it? Is it just a side effect of depression? (P01a)

Bearing out these pre-assessment expectations, in post-assessment interviews, participants strongly emphasised clarification of self-concept in describing the diagnosis’ consequences for their life. Participants described the diagnosis’ effects in terms of ‘explaining myself’ (P05b) and providing ‘an answer about why I am the way I am’ (P12b). This new self-understanding facilitated self-compassion and self-acceptance:

It has meant that I am not a mistake of a person, I am not subhuman, I am not wrong or a freak, for not feeling like the majority of people around me. To be given this is like someone handing me ‘life’. I have been reflecting on suicide since age seven. This has caused a huge shift in how I look at my life. (P13b)

every situation that I go into now, where it’s a struggle [. . .] this makes so much more sense [. . .] I’m autistic. I know I am. Now it’s okay. And I just instantly feel so much better about it. And I don’t feel so lost anymore. (P07b)

#### Anticipation and validation

Pre-assessment, expectations of the diagnosis’ impact varied greatly, from predicting that life ‘will probably continue on in a generally similar manner’ (P12a) to stating ‘it would change everything. It would explain everything. I mean, my whole thirty-two years of existence would be explained in one appointment’ (P07a). Some expected a diagnosis to facilitate concrete advantages, such as ‘the right kind of therapy’ (P11a), ‘qualifying for disability’ (P07a) or making it ‘easier to get accommodations’ (P05a). Others were less optimistic about the likelihood of practical benefits: ‘the daily impact [. . .] would only change if the diagnosis led to helpful resources’ (P04a). Less tangible, but still important anticipated benefits pertained to ‘learning more about myself and what this will mean for self-managing my emotions’ (P12a) and ‘access to a community’ (P03a). Holistically, participants’ investment in the diagnostic process was driven by a desire to move from ‘enduring or tolerating’ (P03a) to ‘live rather than surviving’ (P02a):

if I get this autism diagnosis, it’s kind of the signal that I really need to start accepting these things about myself and learning how to work with them and not against them. (P08a)

In almost all cases, once diagnoses were confirmed, this pre-assessment anticipation turned into post-assessment justification. After diagnosis, most participants described themselves using terms such as ‘satisfied’ (P04b), ‘vindicated’ (P06b) or ‘validated’ (P12b). Since post-assessment interviews were completed shortly after the diagnosis, few participants had yet received resultant practical supports. Nevertheless, the benefits to emotional well-being were sufficient to be characterised as ‘life-changing’ (P05b):

Objectively not much [has changed], but it’s given me far more insight into myself and how I approach the world, and it’s given me a sense of forgiveness for the things I couldn’t do and acceptance of who I am. (P05b)

Pre-assessment, the uncertain status of the diagnosis prompted concern about the ethics of adopting the terminology of autism or ‘appropriating [. . .] this language [. . .] from these communities’ (P03a). One anticipated value of diagnosis was to legitimise use of this linguistic and conceptual ‘shorthand’ (P03a) to describe oneself in personal and interpersonal situations. Post-diagnosis interviews confirmed that participants derived value from having ‘more language to talk to’ (P06b) significant others about one’s challenges. Publicly identifying as autistic cued a host of expectations and attributions, which provided welcome frameworks for contextualising unconventional traits or behaviour. The diagnostic label also worked as a tool for articulating one’s needs and experiences, thereby accessing ‘informal support through discussing my diagnosis with friends’ (P12b).

#### Emotional reflection

Emotionally, the pre-assessment phase was characterised by feeling both ‘excited and nervous’ (P01a). The emotional instability of this ‘limbo’ period represented a mental health risk for some:

it’s also terrifying. I’ve had some of, well, I used to sort of identify as panic attacks. I’ve been as close as I’ve come in many, many years, maybe a decade, by just sort of opening myself to feeling how I feel. And that can be – that opens the dam a lot more, in a lot bigger way than I think I was prepared for or maybe expected. (P09a)

While awaiting assessment, possibility of the diagnosis being withheld was a major worry for some:

I would be gutted. I know myself and I know I would find it incredibly difficult to ask for the things that are currently helping me, if there was no diagnosis of autism. I cannot accept myself for who I am without a professional telling me there is a reason why. I would be devastated and more confused than before this journey of diagnosis began. (P13a)

Relief was almost universally experienced on hearing the diagnosis outcome. One source of relief was relatively positive experiences of the assessment process itself: ‘relief that I didn’t waste the money and that I was being listened to and taken seriously’ (P02a). More fundamentally, relief followed the improvements in self-image brought by the diagnosis: ‘relief to know that there’s a reason that I’m struggling with these things, that it’s not something that I’m doing wrong’ (P11b).

Alongside relief, numerous participants described a post-diagnostic mourning or ‘period of grief’ (P08b) for their previously undiagnosed self: ‘sad as well for the years I’ve lost of myself’ (P05a). Dejection also accompanied the confirmation of deviation from the norm around which society is structured: ‘in the very core of me, in the fabric of who I am, my neurotype is different than the neurotype that all this help was built for’ (P13b).

### Theme 2: Challenges with Navigating the Diagnostic Process

This theme covered the internal and external hurdles that adults faced when engaging with autism assessment.

#### Cognitive overwhelm

Stress was experienced throughout the diagnostic journey. Prior to assessment, participants described themselves as ‘apprehensive’ (P01a), ‘scared’ (P03a) and ‘a bundle of raw nerves’ (P07a). The communication requirements of the assessment itself were a source of anxiety for some:

Quite nervous about being able to express myself properly, answer the questions appropriately, and actually remember examples and situations to accurately present myself during the assessment. (P12a)

Post-diagnosis, numerous participants reported the assessment itself as difficult, describing it as ‘exhausting’ (P10b), ‘stressful’ (P06b), ‘disorienting and very sort of discouraging’ (P09b). Multiple participants mentioned the long duration of appointments, length of forms to complete and sensitive nature of questions asked. The intrusive ‘poking and prodding and kind of felt like a science experiment’ (P07b) nature of the assessment process was ‘draining’ (P02b) and ‘awkward’ (P06b). However, while tiring, intensive assessment procedures also promoted confidence in diagnostic outcomes.

#### Distrust of healthcare systems

Negative experiences of healthcare professionals and systems were pervasive across interviews. For many, the most logistically difficult aspect of the diagnostic journey was the administrative ‘nightmare’ (P03a) of finding a service/specialist who could conduct the assessment:

I had to find all of the facilities myself, I had to hunt down doctors by myself, I had to make all the calls by myself, like they didn’t give me any direction whatsoever. (P07a)

Once assessment was scheduled, apprehension shifted to the possibility of encountering professionals who were ‘dismissive’ (P09a), ‘don’t listen to me’ (P02a), or approached evaluation ‘from a neurotypical perspective’ (P04a). For a minority, such fears were realised, in appointments with ‘sharp’ (P09b) professionals who left them feeling ‘rushed [. . .] vulnerable and exposed’ (P09b). However, positive experiences were more frequently reported with professionals who were ‘personable’ (P07b), ‘open and supportive’ (P05b) and ‘excellent at putting me at ease’ (P13b). Assessment situations that accommodated sensory preferences, through measures such as dim lighting, quiet rooms or online interviews, were particularly frequently praised.

#### Resource constraints

Experiences at all stages of the assessment process were affected by resource constraints. Appropriately qualified specialists were sparse in some areas. High demand resulted in long waits and intensive efforts to schedule assessments:

This meant calling every Monday at 7am. And memorising the phone tree prompts so that I could be the first person in queue to sign up for the newest available opening. (P04a)

Most participants, even in jurisdictions that nominally had publicly-provided assessment pathways, had opted for private assessments, which required major financial outlays:

you’re wondering, like, did I just, you know, set a thousand dollars on fire? (P09b)

While many found the actual assessment to be sensitive and comprehensive, post-diagnosis supports were limited, with the main resources mentioned being information materials and occupational accommodations. After the effort invested in securing and submitting to the assessment, the lack of targeted support could cause dejection:

I wish that the therapies for after the fact were more accessible. Yeah, this part seems harder now than getting in the door with getting a doctor in the diagnosis. So now I’m just like, well, this feels like it’s impossible. And I have to do this by myself too. I’m scared out of my mind, and this feels like an impossible mountain. (P07b)

#### Inadequate consideration of diversity

Throughout the diagnostic process, participants were aware of inequalities of access to and experience in autism assessment. Most participants were women and highly sensitive to the potential role of their gender in delaying their diagnosis to date: ‘there are very few therapists who know about autism in women. It’s almost like we’re a lost cause’ (P11b). While awareness of male-biased assessment methods caused trepidation pre-assessment, no participant reported actual experience of gender bias in post-assessment interviews. Other sources of inequality mentioned were age, income and availability of family members who could complete assessment tools that queried childhood behaviour.

When disclosing their expected or actual diagnosis to others, participants were often gratified by ‘an unspoken acceptance of neurodiversity’ (P04b), with acquaintances affirming the diagnosis ‘makes sense’ (P07a) based on their experience of the person. However, participants believed ‘a lot of people have outdated understanding of what autism entails’ (P03a) and experienced negative stereotypes such as being ‘just lazy’ (P03a) or a ‘princess and high maintenance’ (P02a). Participants had encountered people who ‘profess to be open and welcoming and accepting people, [but] when rubber meets the road, they’re really not that way’ (P04a). As a result, stigma remained a fear and most participants were cautious in disclosing their assessment or diagnosis:

I’m being really selective [. . .] I can’t engage with someone who, you know, this would be the kind of language that I would hear from them. (P03a)

### Theme 3: Isolation to Advocacy

The final theme captures the individual drive required to access an assessment, which could evolve into community support and participation as the diagnostic journey progressed.

#### Self-motivated research

The importance of committed, self-motivated research at the start of diagnostic journeys was clear across interviews. Numerous participants described an ‘aha moment’ (P07a) where they suddenly related the category of autism to themselves. This was followed by intensive reading and engagement with online resources such as screening tests:

I’m going to do a deep dive into this topic and within the next month I had listened to or read probably over a half a dozen books, read tons of articles, and by then there was no doubt in my mind that I’m probably autistic and I decided to seek diagnosis. (P04a)

The information consumed strongly resonated with participants, who became convinced in its applicability to themselves:

the more that I read, and the more that I watch, and the more that I engage with, I’m like, yup, that’s me, yup, that’s me, yup, that’s me. (P06a)

it was like discovering an ancient city in an archaeological dig, the more I read the more I saw and the more into my past and present self the more I saw. (P13a)

#### Guidance from the autistic community

In pursuing their independent research, people benefitted directly and indirectly from support offered by the autistic community. Vicariously ‘reading other people’s experiences online’ (P06a) revealed the diversity of autistic lived experiences, proving a ‘huge help in terms of learning how autism may present itself in various people’ (P12a). Realisation that they ‘identify a lot with characters that were on the spectrum’ (P02a) strengthened participants’ suspicions that they themselves ‘could very well be autistic too’ (P11a). Self-identification as neurodivergent prompted adults to seek relationships with similar others, who validated their new identity:

I made some friends with people who were autistic [. . .] and it was like yeah, I fit in here, I understand their experiences. (P01a)

Existing relationships were also a source of shared identity and solidarity, as well as practical guidance along the diagnostic journey:

I have also been EXTREMELY fortunate to have a good friend who has been down this path already, and was able to help me navigate the process to seek diagnosis. (P12a)

Many participants in the pre-assessment interviews were already deeply invested in neurodivergent communities. However, others remained reticent and awaited the diagnosis to ‘feel more comfortable joining into neurodivergent groups’ (P10a) or acquire ‘the confidence to go out and maybe meet other people more like me’ (P14a).

#### Mentorship and campaigning

After diagnoses were formally confirmed, several participants expressed interest in ‘giving back’ to the autistic community by sharing their experiences or advocating for better supports. For instance, one participant described making a social media post sharing their diagnosis and inviting questions, though with the caveat that, ‘I don’t know if I have the answer. I don’t know if I have the energy to run you through the past year of research that I’ve done to explain this’ (P09b).

While opportunity for concrete acts of activism or advocacy was limited by the recency of participants’ diagnosis at time of interview, entering this space was an intention for many:

I’m still in the stage of like wrapping my head around it and taking the label, and like applying it to experiences it in my life. But I do think that I’ll get to the point where I’m gonna be willing to share and be vocal about it. (P06b)

## Discussion

With growing numbers of adults seeking and receiving autism diagnosis, this study provides novel insight into adults’ evolving experiences throughout the diagnostic process. The analysis revealed the diagnostic journey as rooted in a drive for self-understanding, propelled by anticipated benefits that were partially realised, and emotionally complex at all stages. Acquiring and adjusting to a diagnosis was challenged by issues of resource access, system deficiencies, social inequalities and individual overwhelm. Yet the transformative nature of the diagnosis rendered this challenging trajectory worthwhile for most, particularly by steering people from individual isolation into community participation and advocacy.

Much previous evidence has highlighted gaps in service provision after an adult autism diagnosis (Norris et al., 2025; Scattoni et al., 2021; Wigham et al., 2023). This study’s accounts from post-diagnosis adults across multiple jurisdictions affirmed this experience, with few reporting being offered targeted supports. However, the triangulation of pre- and post-assessment data suggests that for autistic adults themselves, practical supports may not be foremost in determining the subjective value of a diagnosis. While some participants were hopeful of specific accommodations, others did not anticipate tangible benefits. Indeed, the intensive research necessary to secure an assessment meant all entered the process highly aware of deficiencies in service provision and support. Nevertheless, the assessment was still deemed valuable for its role in resolving long-held questions about one’s own identity and behaviour. The post-diagnosis interviews confirmed that this anticipated value was realised: all participants attributed to the diagnosis improvements in self-understanding and self-esteem.

An important insight from the two-stage data collection is that while some participants had begun to apply the lens of autism to understand themselves prior to assessment, this was tinged by doubt and self-criticism. With their pre-assessment research rendering them highly sensitive to the injustices perpetrated against this marginalised community, adults worried about the ethics of ‘appropriating’ autistic language and entering autistic communities before formal diagnosis. Thus, though self-diagnosis can fill important psychological functions (Lewis, 2016; Overton et al., 2024; Sarrett, 2016), formal diagnosis remains necessary for comfort and confidence adopting an autistic identity.

This said, in a context of gaps and delays in access to assessment, it is important to note that certain informal supports were not contingent on diagnosis. All adults described an early research phase that, while undertaken alone, involved resources developed or disseminated by autistic communities. Through consuming freely available accounts of autistic experiences, participants both acquired factual information about autism diagnosis and commenced a psychological identification with the autistic community. While this identification sharpened post-diagnosis, peaking in embarkment on advocacy and activism, community engagement had begun prior to diagnosis, with numerous participants in the pre-assessment interviews already deeply invested in neurodiversity networks. With autistic social identity a known contributor to emotional well-being (Cage et al., 2022; Cooper et al., 2017), these community resources represent a key vehicle of support for adults at early stages of the diagnostic journey.

Identification of such vectors of support is important, given the challenges of the pre-assessment period revealed by this analysis. Adults on waitlists are not typically designated a population in need of targeted support. However, the data showed that the process of securing and awaiting autism assessment can be extremely stressful, compounding the mental health challenges already faced by many in this population (Lai et al., 2019; A. J. Russell et al., 2016). Prior research, consistent with the post-assessment interviews in this study, indicates that responses to a diagnosis are emotionally complex, with relief comingling with grief and despair (Nayyar et al., 2025). A novel contribution of this study is to show such emotional ambivalence also characterises the pre-assessment period, with excitement about anticipated answers mixed with apprehension about the assessment itself and its outcomes. Support navigating this emotional ambiguity would help prepare adults for the demands of the assessment process and its aftermath.

Other practical implications arising from the data pertain to the structure and delivery of assessment itself. Echoing other research across jurisdictions (Huang et al., 2020; Kiehl et al., 2024; Nayyar et al., 2025), the data indicate that adults would benefit from more streamlining and transparency around clinical/self-referral pathways, as well as efforts to tackle the high financial costs involved. Indeed, the current data likely understate the administrative and financial barriers to assessment, given that inclusion criteria meant all participants had successfully surmounted them. Attention should also be given to the cognitive, emotional and sensory demands of how assessment procedures are delivered, which formed a major source of apprehension prior to assessment. However, numerous adults were pleasantly surprised by the neurodiversity-affirmative tone of communications and sensory accommodations made to the assessment environment. Divulging more specific information about such positive service elements prior to assessments would be a low-cost way of mitigating some of the anxiety experienced leading up to appointments.

### Limitations and future research

Contributions of this study should be considered in light of several limitations. Although a large amount of data was analysed, this came from just 14 individuals, with 12 completing both interviews. While this sample size is not unusual for qualitative research on lived experiences of adult autism (Huang et al., 2020; Kiehl et al., 2024; Nayyar et al., 2025), further research is needed to determine whether observed patterns transfer to other contexts. Reliance on opt-in recruitment may have attracted those with higher investment or interest in their diagnosis: more purposive sampling techniques, for example by randomly selecting people from existing waitlists, could yield greater diversity of perspectives. Greater sociodemographic diversity would also be beneficial: this sample was dominated by women in their twenties or thirties, from wealthy, Westernised and predominantly English-speaking countries. While inclusion of multiple countries revealed how patterns in autistic experiences can transcend jurisdictional disparities, the low numbers of participants from individual countries made it impossible to systematically analyse how lived experiences were contingent on regional variability in health systems or service provision. A further study limitation is the absence of participant ethnicity or sociodemographic data. While detailed subgroup analysis was not a focus of this small-scale qualitative study, understanding how assessment experiences intersect with other dimensions of inequality is a key priority for future research. Importantly, study inclusion criteria that required having a scheduled autism assessment precluded those who did not have the social, psychological or material resources to reach this stage. The experiences of those who have tried and failed to access autism assessment, who likely represent a particularly vulnerable cohort, are an important focus for future research.

Participants in pre-assessment interviews described fear and anxiety about the chance of their assessment *not* producing an autism diagnosis, despite their heavy investment in this identity. The current study does not shed light on this outcome, since all participants who completed post-assessment interviews had received an autism diagnosis (the diagnostic status of the two individuals who were lost from the post-assessment interviews unfortunately remains unknown). Having a diagnosis withheld or disconfirmed may be a challenging experience, given most participants’ heavy emotional and financial investment in securing an assessment, and numerous people’s pre-assessment absorption of autism into their self-concept. Future research should aim to capture experiences of the diverse outcomes that clinical assessments can produce.

Serial interviews are an under-used approach in qualitative research (Murray et al., 2009; Read, 2018) and proved effective at tapping the evolution of experiences across the diagnostic journey. However, restricting interviews to within six weeks either side of assessment limited the information gathered: for example, the details of post-diagnosis service provision were not yet clear for some participants, and some had not completed or even begun the process of communicating their new diagnosis to others. The data were also analytically challenging, given the limited availability of practical guidance on conducting longitudinal qualitative analysis (Hermanowicz, 2013). The approach adopted, whereby each interview-pair was analysed as a single case, worked well in answering the study’s aims, but other approaches to the data (e.g. analysing the pre- and post-assessment interviews as two separate datasets) may have yielded different insights. Further analytic complexities related to the multiple interview modalities, although the choice of videocall or email interviews promoted authenticity of data by accommodating diverse communication and sensory preferences.

Providing original evidence of how lived experiences of adult autism diagnosis evolve across the diagnostic journey, the current study offers valuable context for adults pursuing assessment, professionals performing assessments, and policy-makers designing adult autism services. Results highlight the fortitude and initiative of late-diagnosed adults, who have surmounted many barriers to acquire their diagnosis. The journey to diagnosis can be lengthy and beset with administrative, emotional and social obstacles. To date, the work of addressing many of these problems has fallen to the autistic community itself, which has rallied to the challenge of supporting and advocating for those who have yet to formally join its ranks. Properly resourced support for the growing number of adults awaiting autism assessment should be a priority for future research and policy.
